# Mechanisms of PARP1 inhibitor resistance and their implications for cancer treatment

**DOI:** 10.1093/narcan/zcac042

**Published:** 2022-12-22

**Authors:** Lindsey M Jackson, George-Lucian Moldovan

**Affiliations:** Department of Biochemistry and Molecular Biology, The Pennsylvania State University College of Medicine, Hershey, PA 17033, USA; Department of Biochemistry and Molecular Biology, The Pennsylvania State University College of Medicine, Hershey, PA 17033, USA

## Abstract

The discovery of synthetic lethality as a result of the combined loss of PARP1 and BRCA has revolutionized the treatment of DNA repair-deficient cancers. With the development of PARP inhibitors, patients displaying germline or somatic mutations in BRCA1 or BRCA2 were presented with a novel therapeutic strategy. However, a large subset of patients do not respond to PARP inhibitors. Furthermore, many of those who do respond eventually acquire resistance. As such, combating *de novo* and acquired resistance to PARP inhibitors remains an obstacle in achieving durable responses in patients. In this review, we touch on some of the key mechanisms of PARP inhibitor resistance, including restoration of homologous recombination, replication fork stabilization and suppression of single-stranded DNA gap accumulation, as well as address novel approaches for overcoming PARP inhibitor resistance.

## Graphical Abstract

**Graphical Abstract:**
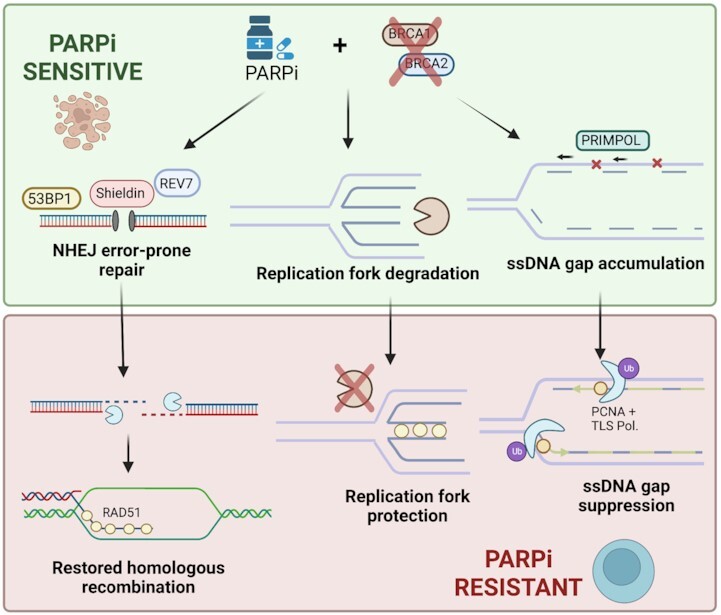
Cancer cells deficient in BRCA1/2 are sensitive to PARP1 inhibitors due to error-prone DNA repair, vulnerable replication forks and single-stranded DNA gap accumulation. Here, we discuss cancer resistance to these drugs.

## INTRODUCTION

Back in 2005, two groups set out to determine whether cancer cells deficient in homologous recombination (HR) could be specifically targeted in a synthetically lethal manner. This led to the discovery that cells deficient in breast cancer gene 1 (BRCA1) or 2 (BRCA2) rely heavily on the single-strand break repair protein poly(ADP-ribose) polymerase 1 (PARP1). In accordance with this, BRCA and PARP1 were found to be synthetic lethal with each other (1,2), and a race to get PARP1 inhibitors (PARPi) to the clinic began. Almost 20 years later, this discovery has revolutionized the way we approach cancer treatment. Patients harboring BRCA-mutant cancers showed a dramatic improvement in tumor response upon the introduction of PARPi to the clinic. While there are at least 17 proteins within the PARP family, the structures and in particular the functions of PARP family members can vary widely (3). PARP1 and PARP2 have been studied extensively for their roles in DNA repair. There are currently a number of clinical PARPi, including olaparib, niraparib, rucaparib and talazoparib, that have potent and selective catalytic inhibitory activity against PARP1 and PARP2, and are used in cancer treatment (4). However, as with many targeted therapies, acquired resistance to PARPi poses an obstacle to achieving durable responses in patients, with tumor recurrence becoming more prevalent. Moreover, a large subset of patients show *de novo* resistance and therefore never respond to PARPi (5). As such, identifying mechanisms of resistance to PARPi and then developing approaches to target these mechanisms and restore or instill PARPi sensitivity is of critical importance to improve patient outcome.

Because of the role BRCA1/2 play in mediating HR, and the nature of the synthetic lethality between BRCA and PARP1, many early studies of PARPi resistance centered around a restoration of HR as double-stranded DNA breaks (DSBs) were viewed as the sensitizing genotoxic lesion. This led to the discovery of distinct mechanisms of resistance to PARPi *in vitro* for BRCA1-deficient and BRCA2-deficient models, highlighting their divergent roles in HR. Furthermore, later studies revealed that BRCA1/2 are also critical for mediating the protection of reversed replication forks (RFs) upon encountering replication obstacles. Subsequently, further investigations revealed that restored RF protection and stabilization also promotes PARPi resistance in BRCA-deficient cells. Recently, some groups have challenged the model in which failure to repair DSBs defines sensitivity to PARPi in HR-deficient (HRD) cells. Instead, it has been proposed that accumulation of single-stranded DNA (ssDNA) gaps is the primary genotoxic lesion promoting PARPi sensitivity and suppression of these ssDNA gaps promotes PARPi resistance. While the focus of this review is on BRCA-mutant cancers, it is also important to note that BRCA wild-type (WT) and HR-proficient cancers have been shown to respond to PARPi as well, due to stabilization of PARP1 on DNA upon PARPi treatment, a process known as PARP trapping, forming replication obstacles (4). In this review, we cover mechanisms of PARPi resistance, including restoration of HR via loss of non-homologous end joining (NHEJ), Polθ-mediated end joining (TMEJ), BRCA1/2 reversion mutations, epigenetic modifications and increased expression of HR factors. We also touch on RF stabilization, either through loss of fork reversal or through protection of reversed forks from degradation as a mechanism that promotes PARPi resistance in BRCA-deficient cells. Additionally, we discuss the new shift in the field centered around ssDNA gaps and the implications of gap suppression in PARPi resistance. Finally, we touch on approaches for overcoming PARPi resistance in the clinic.

## RESTORATION OF HR

Due to the well-established roles of BRCA1/2 in HR DSB repair, the PARPi hypersensitivity of BRCA1/2-deficient cancer cells was originally proposed to reflect their synthetic lethality. In this model, a single-strand break in PARPi-treated cells is unable to be repaired by PARP-mediated base excision repair and is therefore converted to a DSB during DNA replication. This DSB is left unrepaired or forced down the error-prone NHEJ repair pathway or alternative end joining pathways in HRD BRCA-mutant cells leading to genomic instability and subsequent cell death (1,2). In line with this mechanism of sensitivity, a commonly observed mode for acquired PARPi resistance is restoration of HR. Mechanisms of HR restoration include loss of NHEJ factors, stabilization of mutant BRCA1, BRCA1/2 reversion mutations and increased expression of other HR factors.

### Loss of NHEJ factors in BRCA1-deficient cells

**Figure 1.**
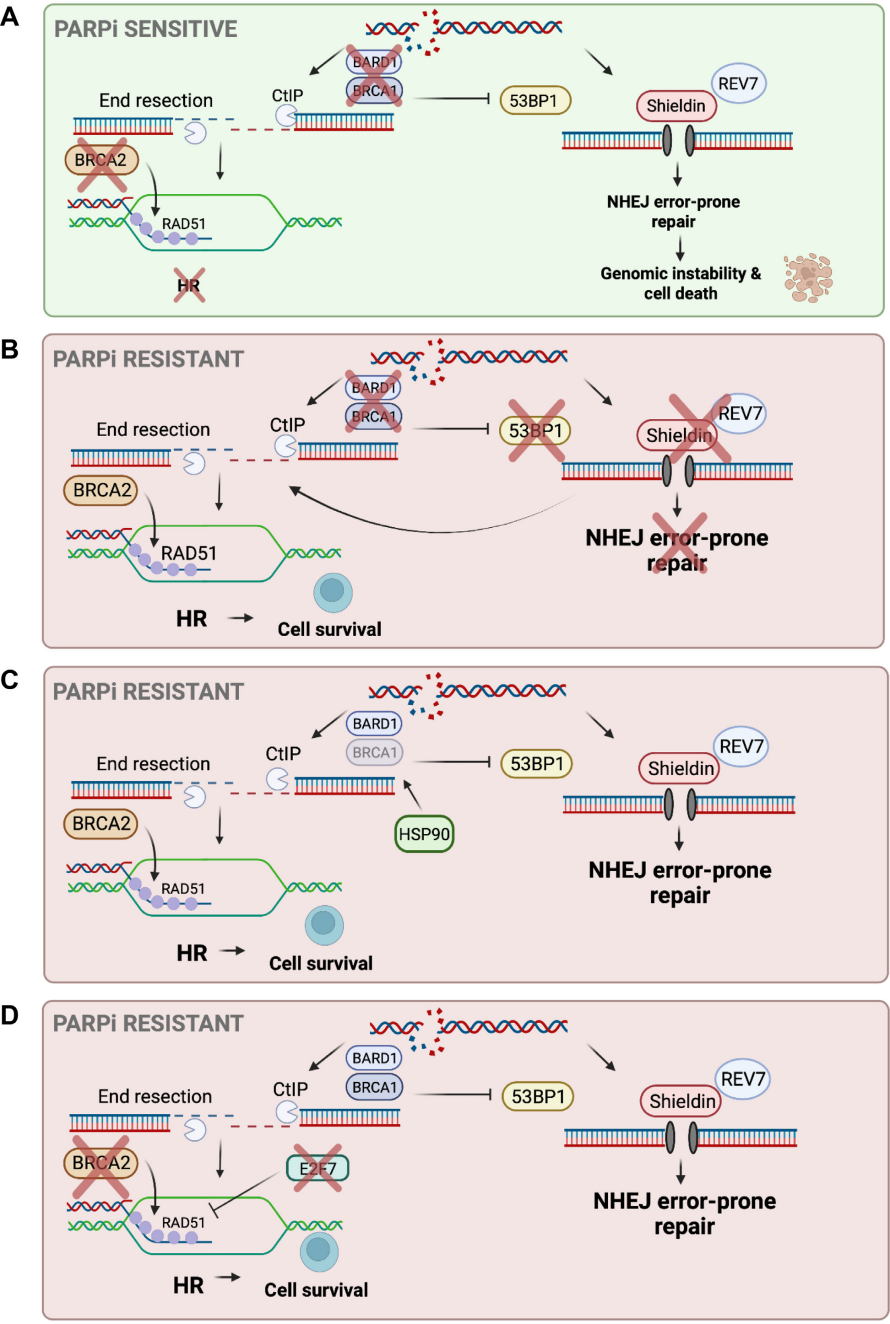
Restoration of HR promotes PARPi resistance. (**A**) Loss of BRCA1 or BRCA2 renders a cell deficient in HR and reliant on error-prone NHEJ to repair break-inducing lesions. This leads to genomic instability and sensitivity to PARPi. (**B**) In BRCA1-deficient cells, loss of NHEJ factors such as 53BP1 or REV7 allows nucleases to participate in end resection and HR to go on in a BRCA1-independent manner leading to PARPi resistance. (**C**) Mutations leading to improper folding of BRCA1 can be stabilized by protein chaperones including HSP90, which promotes restored HR and PARPi resistance. (**D**) Increases in other HR factors such as RAD51 can promote HR despite loss of BRCA2. E2F7 transcriptionally represses RAD51 and loss of E2F7 was shown to increase RAD51 levels and restore HR in BRCA2-deficient cells leading to PARPi resistance. Created with BioRender.com.

NHEJ, an error-prone repair pathway, can function in any phase of the cell cycle, while HR, an error-free repair pathway, is limited to late S and G2 phases of the cell cycle due to the necessary availability of sister chromatids. NHEJ and HR therefore exist in a constant state of competition for repairing DSBs that occur in S and G2. While both BRCA1 and BRCA2 are critical for HR, their roles are distinct, which leads to separate mechanisms of resistance that give rise to restored HR function. While BRCA2 appears to be crucial for loading of RAD51 on the RPA-coated 3′ overhangs, BRCA1, upon association with its obligate binding partner BARD1, has been shown to prevent 53BP1–RIF1 binding at the DSB, thus preventing NHEJ-mediated repair in S and G2 and committing the cell to HR (6–8) (Figure 1A). It was recently shown that BRCA1’s function in blocking NHEJ is limited to S and G2 due to its necessary recognition of histone H4 unmethylated at lysine 20 (H4K20me0). H4K20 methylation status oscillates throughout the cell cycle with its completely unmethylated state marking its incorporation into newly synthesized DNA, thus signaling the availability of sister chromatids (6,9). The ankyrin repeat domain of BARD1 was identified as the reader of H4K20me0, a critical step for HR with potential implications in PARPi resistance (6). However, much still needs to be investigated to fully understand the ramifications of the contributions of this BARD1 domain to PARPi sensitivity.

Due to the role of BRCA1 in blocking NHEJ and committing the cell to HR, it was shown that loss of BRCA1 can be overcome by concomitant loss of NHEJ factors such as 53BP1, thus leading to PARPi resistance in BRCA1-deficient cells (10–12). A major distinction between NHEJ and HR is that NHEJ can take place with minimal end processing, unlike HR that requires extensive end resection at the site of the DSB. 53BP1 works with SHLD3 and RIF1 to recruit the shieldin (SHLD) complex to DSBs to prevent end resection, thus committing the cell to NHEJ DSB repair (13,14). Therefore, in BRCA1-deficient cells, loss of 53BP1 impairs the recruitment of the SHLD complex to DSBs, which allows DSB end resection by nucleases such as MRE11, CtIP, EXO1 and DNA2, and subsequent BRCA1-independent HR (Figure 1B).

Furthermore, it was shown that loss of other NHEJ factors such as REV7, a downstream effector of 53BP1, confers PARPi resistance in BRCA1-deficient cells. In 2015, Xu *et al.* performed an shRNA loss-of-function screen in cell lines derived from BRCA1^−/−^p53^−/−^ mouse mammary tumors to identify BRCA1-independent mechanisms leading to restored HR (15). Among the top hits of the screen, including 53BP1, was REV7. It was revealed that upon loss of REV7, CtIP-mediated end resection is re-established, therefore facilitating restoration of HR and PARPi resistance, which can be reversed by ATM inhibition (15). REV7 is classically known for its role in translesion synthesis (TLS), where it interacts with REV1, and together with the catalytic subunit REV3 forms the TLS polymerase ξ (16). This raised the question of whether REV1 and/or REV3 are also implicated in PARPi resistance or whether this is a REV7-specific phenotype. In contrast to REV7, depletion of REV1 or REV3 showed no significant PARPi resistance. With the use of GFP-tagged REV7, it was observed that following DNA damage, 53BP1 and REV7 colocalize to the site of damage (15), further supporting a REV7 role in mediating PARPi resistance, distinct from its role in TLS.

Importantly, restoration of DNA end resection leading to restored HR function has been identified as a mechanism of PARPi resistance in BRCA1-deficient cells but not BRCA2-deficient cells (10–12,15). This supports their established divergent roles in HR and exemplifies a mechanism by which cells can bypass BRCA1-deficient vulnerabilities by re-establishing HR in a BRCA1-independent manner. In contrast, we recently showed that in BRCA2-deficient cells loss of TIP60 (also known as KAT5) leads to an increase in 53BP1 engagement at DSBs and subsequent PARPi resistance. This suggests that in a BRCA2-deficient background, increasing rather than preventing NHEJ can promote resistance (17). This contrast likely reflects the mechanistically differing roles of BRCA1 and BRCA2 in HR-mediated DSB repair.

### Polθ-mediated end joining and PARPi sensitivity

Beyond HR and NHEJ, under certain conditions DSBs can also be repaired by two other processes, namely single-strand annealing (SSA) and alternative end joining, also known as TMEJ. Like HR, they require DNA end resection to generate 3′ ssDNA overhangs. Importantly, this resection generates the required substrate for HR, SSA or TMEJ while also preventing NHEJ (18,19). In TMEJ, following RPA displacement from the 3′ ssDNA overhangs, Polθ is recruited, and end pairing and microhomology searches take place, followed by trimming of the 3′ DNA tails and DNA synthesis to resolve the break. While the intricacies of this repair pathway remain to be defined, Polθ recruitment has been shown to be critical and it is believed that this depends on PARP1 (19,20). PARP activity promotes the formation of Polθ foci as well as the colocalization of Polθ with CtIP, an important activator of end resection. Furthermore, it was shown that loss of PARP activity partially inhibits TMEJ *in vitro* (20), suggesting that TMEJ is a PARP1-dependent process.

Because TMEJ serves as an alternative pathway to deal with DSBs, cells deficient in HR such as BRCA-mutant cells can become dependent on TMEJ for repair of DSBs (19,21,22). This is particularly important for HRD cells that additionally display loss of NHEJ factors such as 53BP1 or members of the SHLD complex. Indeed, it has been shown that NHEJ can compensate for loss of TMEJ (20) showing the dynamic balance of these various repair pathways. Importantly, it has been shown that inhibition of Polθ is synthetic lethal with loss of BRCA and can synergize with PARPi treatment. Moreover, defects in NHEJ that elicit PARPi resistance in BRCA-deficient cells, such as those described above, can be re-sensitized upon treatment with a Polθ inhibitor (22), suggesting that Polθ and the TMEJ pathway have important implications for the treatment of BRCA-mutant cancers. Indeed, the Polθ inhibitor ART4215 is currently being evaluated in a global open-label phase 1/2 clinical trial in patients with BRCA-deficient breast cancer as a monotherapy or in combination with the PARPi talazoparib and niraparib (NCT04991480). Recently, Heijink *et al.* reported that PARPi treatment leads to under-replicated regions of DNA, which, in mitosis, promote sister chromatid exchange (SCE) independent of HR status. However, in the absence of HR, this can leave behind DNA lesions such as DSBs and ssDNA gaps. The authors identified a role for Polθ in the repair of these mitotic DNA lesions in BRCA2-deficient cells as depletion of Polθ in these cells led to a decrease in SCEs. Furthermore, treatment of these cells with a Polθ inhibitor resulted in chromosome fragmentation, indicative of defective processing of mitotic breaks derived from under-replicated DNA (23). This role for Polθ in mitotic processing of stalled RFs further supports the synthetic lethal relationship between BRCA and Polθ.

Recent reports suggest that Polθ inhibitors may serve an important role in cancer treatment beyond just HRD cancers. DNA repair inhibitors including those targeting NHEJ via DNA-dependent protein kinase (DNA-PK) inhibition are currently being investigated for the potential to sensitize cancer cells to chemotherapies (24–26). Patterson-Fortin *et al.* recently reported a genome-wide CRISPR knockout screen to identify genes that regulate the response to the DNA-PK inhibitor peposertib, which identified Polθ, among other TMEJ genes, to be a key predictor of the response to DNA-PK inhibition. Combined inhibition of DNA-PK and Polθ was found to be synthetic lethal and promoted pathological DSB end resection. Importantly, they found that TP53-mutant cancer cells that displayed resistance to peposertib were sensitized by treatment with a Polθ inhibitor (26). Taken together, these findings suggest that Polθ inhibitors have the potential to improve patient response to therapy in combination with various targeted approaches in specific genetic backgrounds, including PARPi treatment in HRD cancers and NHEJ inhibition in TP53-mutant cancers.

### Stabilization of mutant BRCA1

In contrast to reinstating HR despite BRCA1 deficiency, mutant BRCA1 can also be re-activated, thus promoting critical interactions with HR factors and PARPi resistance. BRCA1 harbors multiple important domains for protein interactions, including the BRCA C-terminal domain (BRCT), which is critical for ensuring proper protein folding. Cells with mutations in this domain exhibit increased vulnerability to protease-mediated degradation (27–29). This ultimately leaves cells deficient in HR and sensitive to DNA damaging agents and PARPi. It was previously shown by Johnson *et al.* that PARPi-resistant, triple negative breast cancer (TNBC) cells with BRCA1 harboring a truncated BRCT co-immunoprecipitated with HSP90, a protein chaperone that assists with protein folding and stabilization (29). The stabilized mutant BRCA1 could interact with PALB2–BRCA2–RAD51 to promote HR and PARPi resistance. Furthermore, it was revealed that treatment of these cells with an HSP90 inhibitor reduced levels of mutant BRCA1 and sensitized the cells to PARPi suggesting that HSP90 can stabilize BRCT mutant BRCA1 promoting restoration of HR and PARPi resistance (29) (Figure 1C).

### BRCA1/2 reversion mutations

In the clinic, commonly seen mutations in BRCA1/2 that abolish HR are single-nucleotide mutations or small insertions or deletions that lead to a shift in the reading frame. This leaves the possibility of secondary reversion mutations that restore the reading frame of BRCA1/2, thus restoring HR activity and conferring PARPi resistance. Indeed, this is observed in patients harboring mutant BRCA1/2 and who are therapy resistant (30–33). Using CAPAN1 cells, a pancreatic cancer cell line harboring mutant BRCA2, it was previously shown that cisplatin selection can promote secondary mutations that lead to a restoration of the BRCA2 reading frame and subsequent resistance to cisplatin and PARPi (34). Furthermore, another group using the same CAPAN1 cell line generated PARPi-resistant clones through PARPi selection and revealed a deletion of the BRCA2 mutation, which allowed these clones to form DNA damage-induced RAD51 foci and reduced genome instability suggestive of intact HR. When BRCA2-deficient cells were reconstituted with the revertant BRCA2 alleles, they exhibited PARPi resistance and HR activity (31).

Acquired PARPi resistance promoted by secondary mutations in BRCA1 has also been shown. Using a breast cancer patient-derived tumor xenograft (PDX) mouse model, prolonged PARPi treatment was administered, and resistant tumors were generated *in vivo* (35). Sequencing of the resistant tumors revealed *de novo* secondary deletions that restored the reading frame of BRCA1. Additionally, immunoblot analysis showed high RAD51 levels, indicative of restored HR. BRCA1-methylated PDX models were also employed and BRCA1 methylation status was assessed in the resistant tumors. A significant portion of the BRCA1-expressing tumors exhibiting therapy resistance showed demethylated BRCA1 promotors and high levels of RAD51 foci suggesting that both genetic and epigenetic modifications can lead to acquired PARPi resistance in BRCA1-deficient tumors (35).

Surprisingly, sequencing of circulating cell-free tumor DNA shows multiclonality of various reversion mutations from a single patient. This exemplifies the intense selection pressure these tumors are under to restore BRCA1/2 function, HR and survive PARPi therapy (36–38). Importantly, reversion mutations in BRCA1/2 may also restore other BRCA-dependent mechanisms, such as RF stabilization, and this alone or in conjunction with HR restoration may confer resistance to other genotoxic agents, including platinum compounds such as cisplatin.

### Increased RAD51 levels in BRCA2-deficient cells

An important HR factor that has also been implicated in PARPi resistance is the recombinase RAD51. Upon DSB formation, RAD51 is recruited and loaded onto RPA-coated ssDNA by BRCA2 and PALB2 where RAD51 protects the 3′ overhangs following resection and facilitates strand invasion. Therefore, in the absence of BRCA2, RAD51 is not able to be efficiently loaded onto the vulnerable resected ends, leading to impaired HR repair of the DSB (39). Our lab recently found that increased levels of RAD51 in BRCA2-deficient cells can lead to PARPi resistance (17,40). E2F7 is a transcriptional repressor that has been shown to target various HR factors, including RAD51 (41). We showed that loss of E2F7 in HeLa BRCA2-knockout (BRCA2^KO^) cells leads to increased RAD51 levels in the cell, which promoted enhanced HR efficiency, RF stabilization and PARPi resistance (40) (Figure 1D). More recently, our lab performed dual genome-wide CRISPR knockout and activation PARPi resistance screens in HeLa-BRCA2^KO^ cells, where the E3 ubiquitin ligase, HUWE1, was identified as a top hit whose loss conferred resistance to PARPi. Interestingly, in HUWE1-knockout cells, unbiased RNA sequencing revealed a roughly 50% increase in RAD51 mRNA expression, which was also associated with an increase in RAD51 protein levels. This corresponded with a significant increase in HR efficiency in HeLa BRCA2^KO^ cells depleted of HUWE1, which likely explains the PARPi resistance observed (17).

An increase in RAD51 has also been identified as a biomarker for PARPi resistance in the clinic. In a 2018 study, PDX from patients harboring BRCA1 or BRCA2 mutations were used to identify *in vivo* mechanisms of PARPi resistance (42). An increase in RAD51 foci was identified in both PDX and patient samples with either primary or acquired PARPi resistance. In most cases, this increase in RAD51 foci was not accompanied by reversion mutations in BRCA1/2 (42). This correlation between RAD51 foci and PARPi resistance in PDX and patient samples supports a mechanism whereby increased RAD51 can restore HR regardless of BRCA status, thus promoting PARPi resistance, and provides clinical relevance to this mechanism.

## RF STABILIZATION

**Figure 2.**
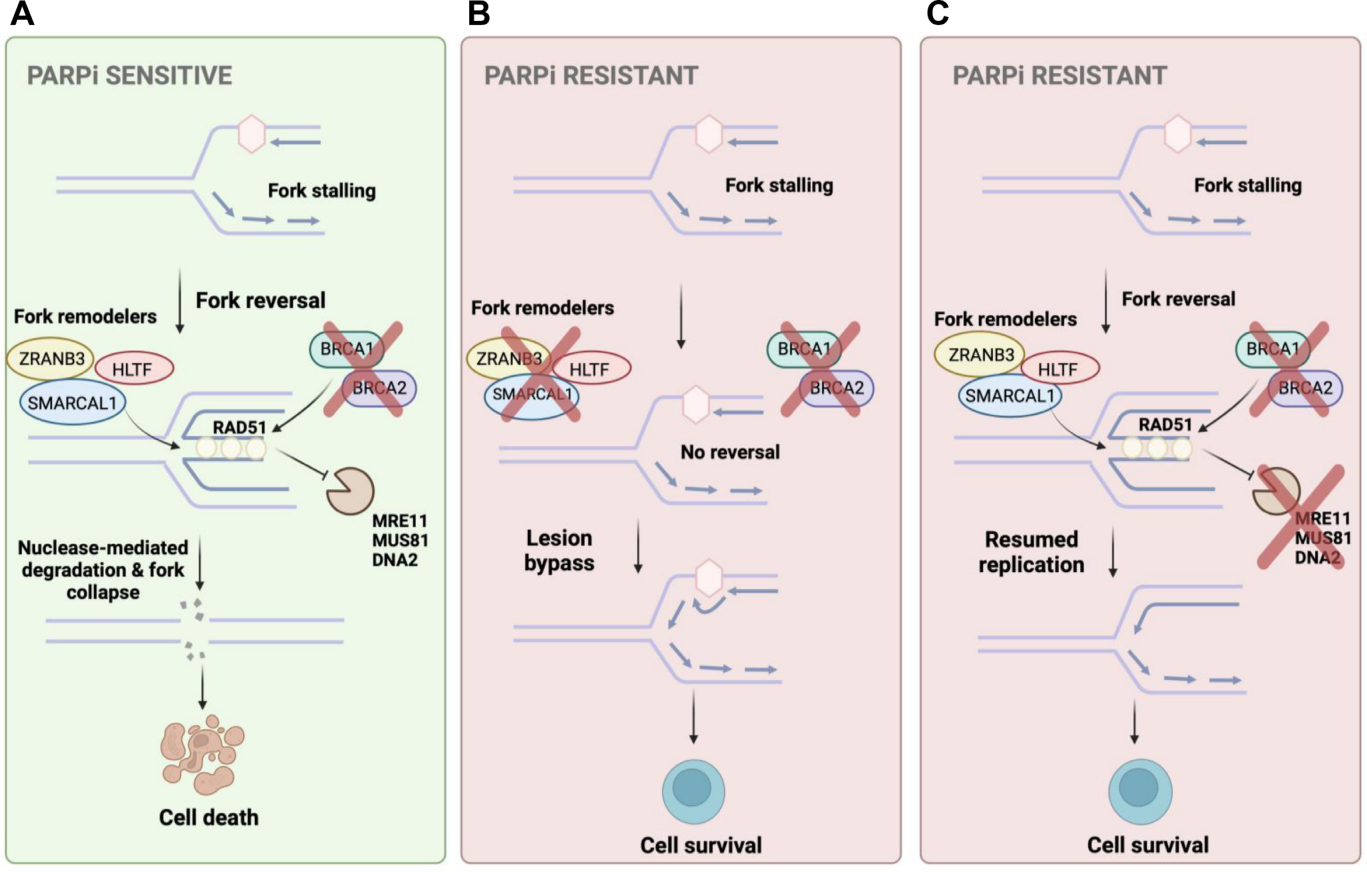
RF stabilization confers PARPi resistance regardless of HR status. (**A**) Replication obstacles lead to fork stalling and reversal so that the lesion can be dealt with. Fork reversal leaves a structure reminiscent of a DSB that is vulnerable to nucleolytic degradation. In BRCA-deficient cells, the reversed structure is unable to be protected by recruitment of RAD51. This renders BRCA-deficient cells sensitive to replication stress-inducing agents such as PARPi. (**B**) Loss of fork remodelers such as ZRANB3, HLTF or SMARCAL1 prevents stalled forks from being reversed. Thus, in BRCA-deficient cells there is no substrate to be degraded and instead other mechanisms promote lesion bypass allowing for cell survival and PARPi resistance. (**C**) Blocked recruitment of nucleases that mediate fork degradation promotes RF protection and survival in BRCA-deficient cells rendering them resistant to PARPi. Created with BioRender.com.

In addition to their role in HR, it has been established that BRCA1/2 also play a critical role in protecting stalled RFs from nucleolytic degradation (43–45). Upon encountering DNA replication stress of either endogenous or exogenous sources, replication is stalled to allow fork remodelers to come in and reverse the fork leading to annealing of the nascent DNA, creating a four-way junction often referred to as a chicken foot structure. This allows for the source of replication stress to be dealt with before replication is later resumed. However, the reversal and annealing of the nascent DNA strands generates a structure that is vulnerable to nucleolytic degradation and subsequent RF collapse and genomic instability. In order to protect reversed RFs from degradation, BRCA1/2 load RAD51 onto the reversed fork and stabilize the RAD51 nucleofilament, thus preventing nucleases from reaching the fork. Therefore, loss of BRCA1/2 leaves RFs susceptible to degradation, which may potentially sensitize cells to DNA damaging and replication stressing agents (Figure 2A). It has been shown that induction of RF protection, either by preventing fork reversal or by inhibiting recruitment of nucleases, leads to PARPi resistance in BRCA-deficient cells in a manner independent of HR status (43–47). In contrast, it has also been shown that loss of fork protection does not always result in sensitivity to replication stress-inducing agents. In cells harboring the BRCA2 C-terminal mutation S3291A, which disrupts the interaction between BRCA2 and RAD51, thus rendering RFs unprotected, sensitivity to PARPi or HU was not observed (44). The relationship between RF protection and PARPi sensitivity thus remains unclear. While we recently extensively reviewed the determinants of RF stability (48), this section will focus on mechanisms of RF protection in a BRCA-deficient background in the context of PARPi resistance.

### Loss of RF reversal

In eukaryotes, one way to manage stalled RFs is their reversal, in which the nascent strands reverse and anneal to each other forming a four-way junction. This causes a pause in DNA synthesis and allows for the replication block to be removed or bypassed so that replication can continue (47,49). Fork reversal is mediated by fork remodelers, including the SNF2 family members ZRANB3, SMARCAL1 and HLTF (47,50–54). HLTF is a fork remodeler and a ubiquitin ligase that polyubiquitinates PCNA, which leads to ZRANB3 recruitment following fork stalling. RPA-bound ssDNA leads to SMARCAL1 recruitment to stalled forks (47,51,55–58). Loss of SMARCAL1 has been reported to potentially promote PARPi resistance in BRCA-deficient cells (47), and while other SNF2 family fork remodelers have differing substrate specificities, their similar mechanisms of mediating fork reversal (53) suggest their loss might also impact PARPi sensitivity. Conceptually, this can be explained since without fork reversal, there is no substrate for nucleolytic degradation, thus promoting RF protection (Figure 2B). However, further investigation is necessary to make firm conclusions regarding the impact of fork reversal on PARPi resistance. Recent work showed that PARP1 and CARM1 cooperate to promote fork reversal by inhibiting the RECQ1 helicase. Interestingly, CARM1 was shown to be critical for PARP1 engagement with DNA, and loss of CARM1 promoted PARPi resistance, possibly due to a loss of PARP trapping (59). However, whether CARM1 expression levels can stratify patient response to PARPi remains to be investigated.

In a recent report, Taglialatela *et al.* showed that loss of SMARCAL1, ZRANB3 or HLTF stabilized RFs and decreased stress-induced DNA breaks in BRCA-deficient cells (47). Moreover, they found that the fork remodeling activity of SMARCAL1 is critical for MRE11-mediated degradation of reversed forks. Using electron microscopy to directly visualize RFs, it was revealed that loss of SMARCAL1 leads to a reduction in reversed forks similar to BRCA1 depletion; treatment with the MRE11 inhibitor mirin restored the percentage of reversed forks in BRCA1-deficient cells but not in cells co-depleted of BRCA1 and SMARCAL1. Overall, these findings suggest that MRE11 is responsible for degrading SMARCAL1-induced reversed forks. Breast cancer cells depleted of BRCA1 and SMARCAL1 displayed resistance to cisplatin and the PARPi olaparib. Importantly, this resistance was accompanied by RF stabilization detected by DNA fiber assays but not HR restoration as evidenced by a lack of RAD51 foci formation in these cells (47). Interestingly, depletion of just one of the above-mentioned fork remodelers is sufficient to induce the observed fork protection phenotype suggesting that the remaining fork remodelers are unable to compensate for this loss. However, exactly how these fork remodelers cooperate to achieve fork reversal remains unclear although it is thought to be due to the distinct fork substrates they each interact with (47).

### RF protection from degradation

RF protection from degradation upon fork stalling is dependent on BRCA1/2, Fanconi anemia pathway components and RAD51 (45). Using *Xenopus laevis* egg extracts, Hashimoto *et al.* employed electron microscopy to visualize replication intermediates and revealed that RAD51 is critical for protecting nascent DNA from MRE11-mediated nucleolytic degradation (60). However, the role of BRCA1/2 and RAD51 in RF protection has been shown to be independent of HR as loss of other HR components such as RAD54 did not affect RF stability either in the presence or in the absence of HU (44). Moreover, loss of KU70, a key component of NHEJ involved in protecting DNA ends from degradation, also did not lead to a loss of RF protection, suggesting that fork protection is also independent from canonical NHEJ (44). Therefore, while BRCA-mediated RF protection is dependent on RAD51, it is independent of HR (44) indicating that this chemoresistance mechanism is distinct from HR restoration.

Chromatin modifiers have been shown to play a critical role in genomic stability, including in mediating recruitment of nucleases to stalled RFs (61,62). Blocking the activity of nucleases such as MRE11 can promote RF protection in BRCA-deficient cells (44) (Figure 2C). Ray Chaudhuri *et al.* recently reported that loss of PTIP, an MLL3/4 complex component, led to protection of BRCA-deficient cells from DNA damaging agents but not through restoration of HR activity (46). Instead, it was observed that loss of PTIP in BRCA2-deficient cells led to protection, restart and normal progression of RFs following HU-induced fork stalling. This was found not to be the result of RAD51 filament stabilization but instead was due to blocked recruitment of MRE11 (46). MRE11 is a nuclease possessing 3′–5′ exonuclease activity that has been found to degrade nascent DNA at stalled RFs (44,63,64). Classically, PTIP has been shown to be recruited to DSBs by 53BP1 for NHEJ (65); however, the observation that loss of PTIP did not lead to restored HR begged the question of whether PTIP activity at stalled RFs was independent of 53BP1-mediated recruitment to DSBs. PTIP was found to accumulate at stalled RFs in both 53BP1-deficient and WT cells following HU treatment, suggesting recruitment of PTIP to stalled RFs is in fact independent of 53BP1. Furthermore, it was shown that, due to its association with PA1 and the MLL3/4 histone methyltransferase (66,67), PTIP is critical for the deposition of MRE11 on nascent DNA, thus establishing a role for PTIP at stalled RFs. Consistent with this, loss of PTIP in BRCA1-deficient cells was shown to decrease chromosomal aberrations and promote genomic stabilization compared to PTIP-proficient, BRCA1-deficient cells. Furthermore, a query of BRCA1- or BRCA2-mutant ovarian cancer patients from The Cancer Genome Atlas revealed that high PTIP expression correlated with better progression-free survival (PFS) compared to low PTIP expression, which correlated with low PFS. This suggests PTIP expression levels could serve as a biomarker for sensitivity to PARPi in BRCA-mutant patients. Finally, using a KB2P mouse model for BRCA2-deficient breast cancer, long-term PARPi treatment promoted the growth of PARPi-resistant tumors that were found to display RF protection as opposed to HR restoration as the promoting force behind the acquired resistance (46). Altogether these data suggest a model where RF stability can affect PARPi sensitivity independent of HR status in a BRCA-deficient background and that blocked recruitment of MRE11 can contribute to RF protection. In line with this, we recently identified the ADP-ribosyltransferase PARP14 as a novel regulator of MRE11 recruitment to stalled RFs, and showed that loss of PARP14 suppresses MRE11 engagement on nascent DNA in BRCA-deficient cells, and promotes fork protection and resistance to PARPi and cisplatin (68).

The chromatin modifier, enhancer of zeste homologue 2 (EZH2), has also been implicated in PARPi resistance. EZH2 was found to trimethylate histone 3 at lysine 27 (H3K27me3) (69). It has previously been identified as a pro-proliferative oncogene, often amplified in several cancers (70). In contrast, it was recently found that loss of EZH2 can negatively affect chemotherapeutic response in BRCA-deficient cancers. Rondinelli *et al.* reported that EZH2 localizes to stalled RFs to catalyze H3K27me3 formation, which allows for recruitment of the nuclease MUS81 (71). Therefore, loss of EZH2 was shown to prevent MUS81 recruitment to stalled forks, resulting in fork stabilization in BRCA2-deficient cells. Consequentially, this loss of EZH2 in BRCA2-defieicnt cells promoted PARPi resistance. Interestingly, low EZH2 levels correlate with poor survival in patients with BRCA2-mutated cancers, suggesting that EZH2 expression levels, similarly to PTIP, can serve as a biomarker for patient response to PARPi therapy (71). The PAR-binding chromatin remodeler ALC1 has also been identified as a mediator of PARPi toxicity (72–74). PARPi block the catalytic activity of PARP1/2, which promotes trapping of PARP on the chromatin and leads to stalled RFs, fork collapse and DSB formation, which is toxic to HRD cells. ALC1 has been shown to bind PARylated chromatin, subsequently aiding in the removal of inactive, trapped PARP1/2 proteins. In line with this, loss of ALC1 was shown to enhance PARP trapping and promote hypersensitivity to PARPi in BRCA-deficient cells (72–74), suggesting that ALC1 could serve as a potential therapeutic target in BRCA-mutant cancers.

The nuclease DNA2 has also been implicated in PARPi sensitivity in a BRCA-deficient context. Our lab recently showed that PCNA ubiquitination status plays a critical role in DNA2-mediated response to stalled RFs following HU treatment (75). Thakar *et al.* showed that loss of PCNA ubiquitination at K164 leads to nascent strand degradation. Surprisingly, the observed degradation was not found to be the result of MRE11 recruitment to the nascent DNA as treatment with mirin did not suppress the fork degradation phenotype in PCNA-K164R mutant cells. Furthermore, other nucleases including EXO1, MUS81, and CtIP were ruled out as potential nucleases mediating the observed fork degradation. However, inhibition of DNA2 led to a restoration of DNA tract length in the K164R mutant cells suggesting that upon loss of PCNA ubiquitination, DNA2 is responsible for nascent strand degradation. The DNA2-mediated RF degradation was further enhanced in K164R cells deficient in BRCA2. Moreover, cells deficient in BRCA1/2 and harboring the K164R mutation were hypersensitized to PARPi suggesting that PCNA ubiquitination can promote RF protection in BRCA-deficient cells and play a role in determining response to PARPi (75).

In addition to nuclease-mediated degradation of unprotected stalled RFs, factors involved in protection of reversed RFs from nuclease engagement have also been implicated in PARPi sensitivity. Dungrawala *et al.* recently used iPOND coupled with mass spectrometry to identify proteins enriched at stalled RFs (76), where they identified CXorf57 as a protein heavily recruited to stressed RFs and modestly recruited to unstressed forks. This protein, which they named RADX, was found to bind ssDNA and protect stalled RFs by antagonizing RAD51, thus preventing over-recruitment of RAD51 and RF collapse. In contrast, in BRCA2-deficient cells loss of RADX was found to restore RF protection. This was likely due to the loss of RADX’s negative impact on RAD51 recruitment in a way that can compensate for reduced stabilization of RAD51 in BRCA-deficient cells. Importantly, the observed fork protection was achieved in the absence of restored HR. In agreement with this, loss of RADX in BRCA2-deficient cells led to resistance to a variety of replication stressing agents, including the PARPi olaparib. Interestingly, this group found that breast and lung cancer patients with higher RADX expression tend to show better survival than those with low RADX expression, highlighting the clinical relevance of these findings (77).

## ssDNA GAP SUPPRESSION

Recent research in the field is pointing to a potential paradigm shift in the way we view PARPi sensitivity, since new findings argue that ssDNA gaps are the true determining genotoxic lesion as opposed to DSBs (78–80). These ssDNA gaps can derive from a variety of distinct sources and can occur on both the leading and lagging strands. For example, on the lagging strand ssDNA gaps can arise as a result of perturbed Okazaki fragment (OF) processing. However, sources of ssDNA gaps on the leading strand include primase-polymerase (PRIMPOL)-mediated repriming of DNA synthesis upon encountering a replication obstacle. This next section will focus on PRIMPOL-mediated ssDNA gap emergence and the suppression of these gaps as a mechanism of resistance to PARPi in a BRCA-deficient background.

### PRIMPOL-mediated repriming as a replication stress tolerance mechanism

Recent research has led to the emergence of PRIMPOL, a primase-polymerase encoded by the *CCDC111* gene, as a critical player in the response to replication stress *in vitro* (81,82). Since the discovery of PRIMPOL-mediated repriming, there has been intense investigation into pathway choice for dealing with replication stress in a cell. This choice likely depends on the nature of the replication obstacle present, cell cycle phase and the availability of DNA damage tolerance (DDT) factors (83). Upon encountering a replication block, PRIMPOL is able to reprime DNA synthesis downstream of the lesion and resume DNA synthesis. This leaves behind ssDNA gaps that need to be filled, either by TLS or template switching (TS) (83,84). However, improper gap filling, also known as post-replicative repair (PRR), can lead to the persistence of these gaps and subsequent genomic instability.

As discussed earlier, upon encountering a replication obstacle, RFs reverse. However, in BRCA-deficient cells this leads to pathological degradation of the reversed forks. Interestingly, Quinet *et al.* recently reported that PRIMPOL-mediated repriming suppresses fork reversal, thus suggesting a protective role for PRIMPOL in BRCA-deficient cells (85). Using BRCA1-deficient human ovarian cancer cells, they showed that pretreating these cells with cisplatin before adding a challenging dose of cisplatin yielded diminished RF degradation compared to BRCA1-deficient cells not pretreated with cisplatin. They found no increase in TLS polymerases in the BRCA1-deficient cells pretreated with cisplatin but observed a significant increase in PRIMPOL transcript, protein and chromatin-bound levels, which was not observed in BRCA1-proficient cells (85). This suggested that PRIMPOL-mediated repriming could explain the suppressed fork degradation observed in cells pretreated with cisplatin. Indeed, depletion of PRIMPOL in BRCA1-deficient cells pretreated with cisplatin before receiving a challenging dose does restore fork degradation. They went on to determine that it is the primase activity of PRIMPOL (as opposed to the polymerase activity) that suppresses fork degradation in BRCA-deficient cells, leaving ssDNA gaps behind the RFs. Using electron microscopy to visualize the RFs, they confirmed that pretreating BRCA1-deficient cells with cisplatin leads to a decrease in the number of reversed forks (85). Due to the essential role of ATR in maintaining RF stability (86), they went on to determine that upon ATR inhibition, the increase in PRIMPOL levels was abolished, and fork protection observed following pretreatment with cisplatin was diminished similarly to when PRIMPOL was depleted (85). This raises the possibility that the ATR pathway has a degree of transcriptional control over PRIMPOL and is therefore required for PRIMPOL-mediated suppression of fork degradation in BRCA-deficient cells (85). As mentioned earlier, RAD51 and fork remodelers including SMARCAL1 are required for fork reversal to occur. Depletion of either of these factors in BRCA1-deficient cells under the above-mentioned conditions led to ssDNA gap accumulation unless PRIMPOL was also depleted, suggesting that abolished fork reversal promotes PRIMPOL-mediated repriming as a mechanism for addressing replication stress (85).

Kang *et al.* recently reported that BRCA2, along with MCM10, a member of the MCM helicase family, suppresses PRIMPOL repriming and ssDNA gap accumulation (87). BRCA1 and BRCA2 help to restrain RF progression following DNA damage as loss of BRCA1 or BRCA2 was shown to lead to an increase in nascent DNA tract lengths following HU treatment (80) and ionizing radiation (IR) (87). Several studies have shown that BRCA2 is not necessarily required for fork reversal (47,88,89), and Kang *et al.* observed this as well, and reasoned that PRIMPOL could be mediating the unrestrained fork progression observed in the BRCA2-deficient cells due to the findings reported by Quinet *et al.* (85) discussed earlier. They found upon co-depletion of PRIMPOL and BRCA2, the unrestrained fork progression previously observed following IR was completely abolished (87). In accordance with this, an increase in ssDNA gap accumulation was observed in the BRCA2-deficient cells following IR, likely as a result of PRIMPOL-mediated repriming of DNA synthesis. They went on to show that when fork remodelers including ZRANB3, SMARCAL1 and HLTF are co-depleted with PRIMPOL in the presence of IR, there is a significant decrease in fork speed compared to when the remodelers were depleted alone (87). This agrees with the data reported by Quinet *et al.* (85) and further supports the model that, following DNA damage, PRIMPOL-mediated repriming occurs in the absence of fork reversal. Kang *et al.* then went on to show that it is actually BRCA2’s association with MCM10 that suppresses PRIMPOL-mediated repriming. Interestingly, this group reported that co-depletion of BRCA2 and PRIMPOL promoted partial resistance to IR and bleomycin compared to BRCA2 depletion alone (87). This agrees with the emerging model that persistence of gaps determines sensitivity in BRCA-deficient cells, as loss of PRIMPOL should decrease the emergence of ssDNA gaps. However, it is possible that loss of PRIMPOL may disturb DNA replication and cell proliferation in general, which may differentially affect sensitivity to replication-targeting drugs.

The above-mentioned studies established a role for PRIMPOL in dealing with replication stress through suppressing fork reversal and mediating repriming of DNA synthesis downstream of the replication blocking lesion (85,87). Furthermore, it was shown that increasing doses of cisplatin in BRCA-deficient cells leads to increased expression of PRIMPOL and ssDNA gap accumulation, potentially representing a mechanism to prevent fork degradation in these cells (85). However, the exact nature of the balance between the benefits of repriming and when this turns pathological remains unclear but is likely context dependent. This mechanism is particularly relevant to PARPi response in BRCA-deficient cancers as PARPi have been shown to reduce fork reversal (84,90,91), which may further promote PRIMPOL-mediated repriming, potentially tipping the scales to pathological repriming of DNA synthesis. However, this begs the question of how and when PRIMPOL-mediated ssDNA gaps are filled. The PRR mechanism employed could impact the genomic stability of cells as defective gap filling, particularly in BRCA-deficient cells, likely increases chemosensitivity.

To address this gap in the field of gaps, Tirman *et al.* sought to determine how and when ssDNA gaps are filled upon PRIMPOL overexpression or defective fork reversal (84). Over the years, there has been significant debate over which DDT pathways mediate gap filling, with data in bacteria, yeast and human cells suggesting it might be cell cycle dependent. Using these models, it has been suggested that TS, TLS and a RAD51-mediated alternative HR pathway contribute to gap filling; however, the role the cell cycle plays in pathway choice remained largely unknown (84,92–101). To answer this question, Tirman *et al.* employed a kinetic S1 nuclease DNA fiber assay where nascent DNA tract lengths were measured every 4 h following removal of cisplatin over 24 h. They found that by hour 16 the DNA tract lengths in PRIMPOL overexpressing (OE) cells were equal in length to PRIMPOL-OE cells not receiving cisplatin, suggesting that these cells had successfully filled in the ssDNA gaps by 16 h after cisplatin removal. Using EdU cell cycle profiling, G2 was found to be reached at 12–16 h, indicating that by G2 the PRIMPOL-OE cells had repaired their ssDNA gaps. Furthermore, a modified DNA fiber assay to measure PRR revealed a significant increase in PRR tract density in G2/M-arrested PRIMPOL-OE cells 16 h following cisplatin treatment, supporting the previous observation that gaps are filled by G2 (84).

The ubiquitination status of PCNA has been shown to determine whether TLS or TS is engaged for damage bypass. PCNA monoubiquitination at K164 by RAD18 promotes TLS, while PCNA polyubiquitination at K164 by UBC13 promotes TS (84,102–105). We previously showed that PCNA ubiquitination and the BRCA pathway cooperate to suppress ssDNA gap accumulation during DNA replication (75). Indeed, Tirman *et al.* showed that RAD18-promoted PCNA monoubiquitination led to REV1-POLξ-mediated TLS gap filling in G2, while UBC13-promoted PCNA polyubiquitination led to RAD51-depenent TS gap filling in S phase. Moreover, using patient-derived BRCA1- and BRCA2-mutant cell lines it was revealed that combining PARPi with a REV1 inhibitor further sensitizes BRCA-deficient cells (84). This suggests that inhibiting gap filling in BRCA-mutant cells increases their sensitivity to PARPi, possibly due in part to the reduction in fork reversal imparted on the cells by PARPi treatment, leading to the promotion of pathological repriming and gap accumulation.

### ssDNA gap suppression and PARPi resistance

With the explosion of research on the emergence of ssDNA gaps in the field, it begs the question of whether DSBs are the defining genotoxic lesion inducing hypersensitivity to chemotherapy in HRD cancer cells or whether there is a more direct underlying model of sensitivity involving first the generation of ssDNA gaps. The argument that ssDNA gaps define chemosensitivity in BRCA-deficient cells is centered around a few key observations. While BRCA-deficient cells display a defect in the repair of DSBs, they also display defects in suppressing ssDNA gap accumulation upon treatment with a DNA damaging agent (78–80). Indeed, as mentioned earlier, PARPi suppress fork reversal, which promotes repriming and ssDNA gap formation (90,91). Moreover, it has recently been shown that ssDNA gaps can arise in BRCA-deficient cells independent of DSB formation (78,106). In accordance, BRCA-deficient cells displaying chemotherapy resistance have been shown to display suppressed ssDNA gap accumulation linking gaps to therapy resistance (78–80). Finally, in some cases cells with intact HR or fork protection but defective OF maturation display sensitivity to chemotherapies and PARPi (75,78–80), and in contrast, BRCA-deficient cells show resistance regardless of HR or fork protection status, suggesting an incomplete model for chemosensitivity (78,80).

It was previously proposed that chemotherapies such as cisplatin induce DSBs when RFs collide with platinum-induced DNA cross-links, which ultimately leads to fork collapse and formation of DSBs (80,107). However, more recent data suggest that DNA cross-link formation as a result of cisplatin treatment can initially be bypassed without causing fork collapse (80,108,109), suggesting that a different mechanism of cisplatin-induced genomic instability might be responsible for chemosensitivity of HRD cancers. In a recent study, Panzarino *et al.* proposed that chemotherapy-induced genomic instability derives from the accumulation of ssDNA gaps as opposed to defective DSB repair or fork protection. They found that PEO1 BRCA2-mutant patient-derived cancer cells are unable to restrain fork progression in the presence of a low dose of HU. Using a DNA fiber assay modified to detect ssDNA gaps upon S1 nuclease treatment, they found that the BRCA2-mutant cells displayed a significant increase in ssDNA gaps, which can likely explain the unrestrained fork progression (80). Remarkably, it was observed that a variety of genes associated with chemoresistance in BRCA2-deficient cells including CHD4, FEN1, EZH2 and ZFHX3 (46,71,110,111) promoted ssDNA gap suppression in PEO1 cells. In an attempt to uncouple gap suppression from fork protection, Panzarino *et al.* depleted SMARCAL1 to prevent fork reversal, or MRE11, the nuclease responsible for degrading reversed RFs, thus restoring fork protection in these cells; however, ssDNA gaps continued to persist as investigated using the S1 nuclease DNA fiber assay. The overall model proposed was that DSBs are a by-product of programed cell death and that ssDNA gaps represent the true chemosensitivity-defining genotoxic event (80). Separately, it has also been reported that PARPi have a *trans* cell cycle effect on BRCA-deficient cells (112). Simoneau *et al.* recently proposed that PARPi treatment in BRCA-deficient cells induces ssDNA gap formation behind DNA RFs in each S phase of the cell cycle and PARPi-induced PARP trapping prevents proper gap filling. Subsequently, these gaps persist into the next cell cycle and collide with RFs leading to fork collapse and DSBs. Cells with proficient BRCA pathway are able to deal with this stress by suppressing origin firing via ATR and recruitment of RAD51 to repair the DSBs. However, BRCA-deficient cells are unable to suppress origin firing leading to more DSB accumulation. The authors propose that, with each cell cycle, DSBs continue to accumulate in BRCA-deficient cells until a lethal threshold is reached. In this model, ssDNA gaps arise as a result of PARPi treatment; however, the subsequent DSBs ultimately lead to cell death in BRCA-deficient cells (112). In contrast, the model proposed by Panzarino *et al.* posits, as described earlier, that ssDNA gaps promote cell death and DSBs form as a result of apoptosis (80). These divergent models highlight the complexity of defining the determinants of chemosensitivity in BRCA-mutant cells and suggest further investigations are necessary to fully elucidate these aspects.

Recently, Cong *et al.* reported that replication-associated ssDNA gaps determine BRCA–PARP1 synthetic lethality (79). They found that treatment of RPE1 cells with PARPi induces unrestrained fork progression, which agrees with previous reports, but this increased fork lengthening alone does not fully explain PARPi sensitivity as depletion of p21, a cell cycle regulator, in RPE1 cells did not increase sensitivity to PARPi. Comparing a BRCA1-deficient mouse ovarian cancer cell line to one with derived PARPi resistance, it was revealed that the resistant cell line displays suppressed ssDNA gap formation, suggesting that decreased gap accumulation could explain the resistance observed. Furthermore, it was shown that in BRCA1-deficient cells treated with PARPi there was an increase in RPA density at nascent DNA, indicative of an increase in ssDNA. This raises the possibility that initially RPA is recruited to protect ssDNA gaps generated by PARPi in BRCA1-deficient cells; however, once the RPA pool is depleted, gaps persist and genomic instability ensues. Indeed, it was shown that when RPA is depleted, BRCA1-deficient cells become more sensitive to PARPi, but, in contrast, when RPA was overexpressed in BRCA1-deficient cells, PARPi resistance arises. Remarkably, using a Fanconi anemia patient cell line harboring one mutant RAD51 allele that is sensitive to PARPi/cisplatin but proficient in HR, they found an increase in ssDNA gaps that can be restored upon conversion of the mutant RAD51 allele to WT. Even more surprising, depletion of RADX to restore fork protection in this patient cell line did not affect PARPi sensitivity, but accumulation of ssDNA gaps remained, suggesting that persistence of gaps was promoting PARPi sensitivity regardless of HR and fork protection status. The overarching model proposed was that the more ssDNA gaps accumulate, the more sensitive BRCA-deficient cells are to PARPi, and conversely, the less ssDNA gaps present, the more PARPi resistant cells become (79).

Recent studies uncovered a role for PARP1 in mediating OF maturation through a non-canonical OF ligation pathway involving DNA ligase III (LIG3) and XRCC1 (113–115). In the case of BRCA1-deficient cells, Cong *et al.* determined that defective OF processing was a major source of ssDNA gaps with high levels of PARylation. However, in PARPi-resistant cells, PARP1-mediated poly-ADP-ribose (PAR) levels were diminished and OF processing was restored (79). Moreover, our laboratory recently showed that OF processing defects may account for a significant proportion of ssDNA gaps in BRCA-deficient cells, and they drive fork degradation and chemosensitivity (116). Using live cell detection of PAR, it was recently shown that BRCA2-deficient cells have elevated PAR levels, and BRCA2-revertant cells displayed low levels of PAR (117), agreeing with the observations made in BRCA1-mutant cells (79), and suggesting that PAR levels might serve as a biomarker for resistance. Interestingly, Taglialatela *et al.* showed that ssDNA gaps can also accumulate in BRCA1-mutant cells as a result of PRIMPOL-mediated repriming (118). This increase in gaps was exaggerated upon loss of RAD18 (118), which agrees with the idea that RAD18 is critical for inducing PRR of ssDNA gaps (84). Indeed, loss of RAD18 significantly reduced cell viability of BRCA-deficient cells (118). This synthetic lethality was determined to be due to the loss of REV1-mediated TLS to fill in PRIMPOL-dependent ssDNA gaps. It was shown that REV1 inhibition combined with PARPi in BRCA-deficient cells leads to an additive cytotoxic effect compared to either alone (118), agreeing with the findings presented by Tirman *et al.* (84).

It has been revealed that persistence of ssDNA gaps also underlies the synthetic lethality between BRCA and POLθ. Schrempf *et al.* utilized BRCA1-deficient cells to reveal that treatment with a POLθ inhibitor further increases replication stress and ssDNA gap accumulation, suggesting that BRCA1-deficient cells rely on POLθ for processing ssDNA gaps. A genome-wide CRISPR knockout screen identified NBS1, a component of the MRE11–RAD50–NBS1 (MRN) complex, to be critical for DNA damage accumulation in BRCA1-deficient cells treated with POLθ inhibitors. MRN was thus proposed to be responsible for processing ssDNA gaps accumulating under these conditions (119). At the same time, Mann *et al.* reported that BRCA2 synthetic lethality with POLθ is also linked to ssDNA gaps. Using *X. laevis* extracts, they revealed that POLθ aides in processing stalled OFs, thereby promoting ssDNA gap filling on the lagging strand when RAD51 cannot be recruited. Upon POLθ inhibition, ssDNA gaps persist allowing for MRN-mediated cleavage and subsequent RF collapse (120). These recent studies further highlight a role for ssDNA gaps in promoting chemosensitivity in BRCA-deficient cells and support the combined use of POLθ inhibitors with PARPi and other chemotherapies in BRCA-mutant cancers.

**Figure 3.**
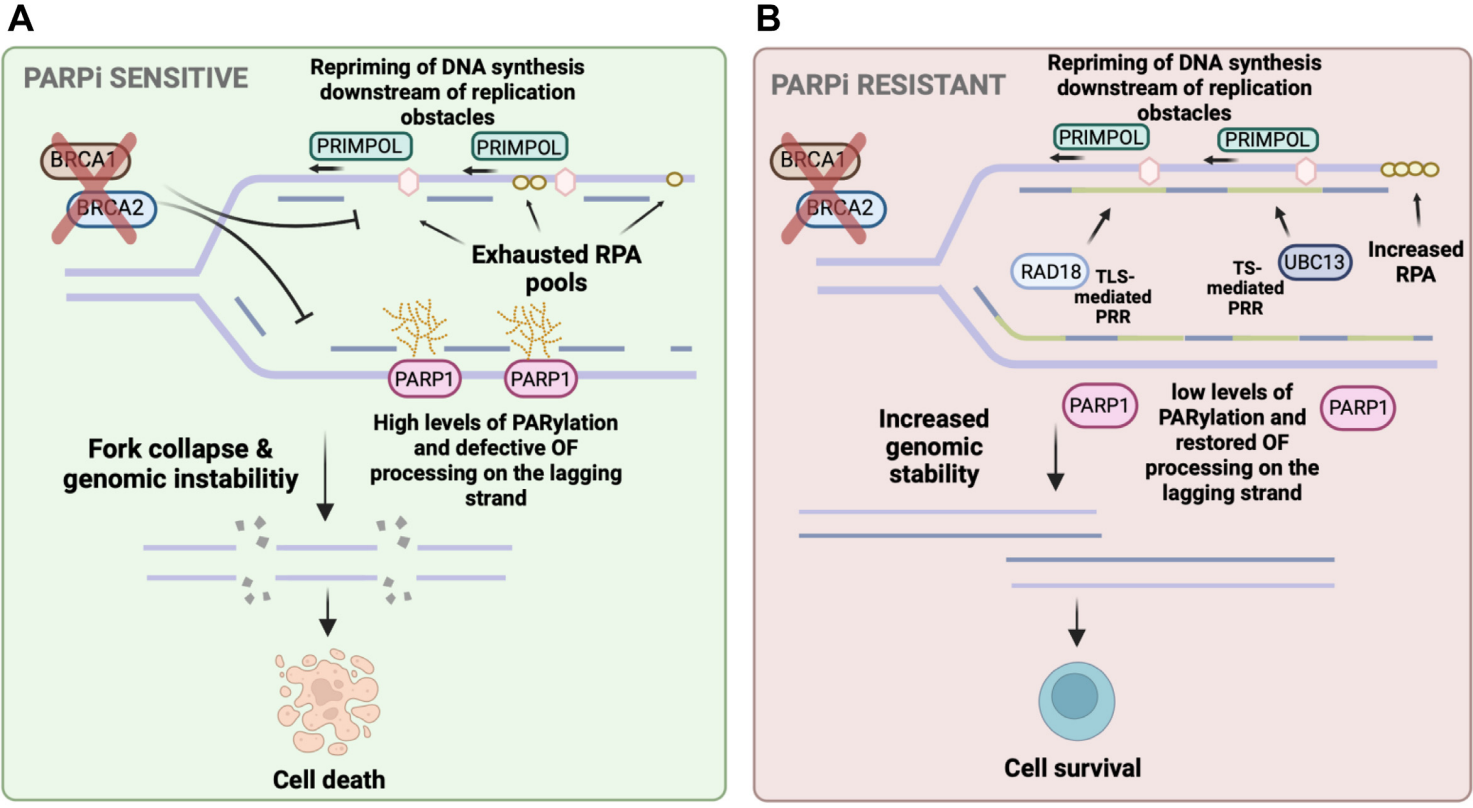
Suppression of ssDNA gap accumulation and PARPi resistance. (**A**) Sources of replicative ssDNA gaps in BRCA-deficient cells include increased PARylation and defective OF processing on the lagging strand, and elevated PRIMPOL-mediated repriming of DNA synthesis past genotoxic lesions accompanied by exhausted RPA pools to protect the gaps and inefficient PRR-mediated gap filling on the leading strand. In the presence of PARPi, this promotes fork collapse, genomic instability and cell death. (**B**) Suppression of ssDNA gap accumulation through decreased PAR levels and restored OF processing on the lagging strand or increased RPA levels, and TLS (promoted by RAD18 monoubiquitination of PCNA in G2)- or TS (promoted by UBC13 polyubiquitination of PCNA in late S)-mediated PPR of the ssDNA gaps on the leading strand promotes increased genomic stability and PARPi resistance. Created with BioRender.com.

Taken together, these finding suggest that ssDNA gaps could serve as a determinant of chemosensitivity in BRCA-deficient cells. In this model, cells lacking functional BRCA1 or BRCA2 would display a decreased ability to suppress ssDNA gaps; therefore, treatment with genotoxic agents such as PARPi would lead to increased ssDNA gap accumulation with diminished ability to protect and fill these gaps, ultimately promoting PARPi sensitivity (Figure 3A). On the contrary, if BRCA-deficient cells acquired an increased ability to protect the ssDNA gaps via increased RPA availability, for example, or if these cells acquired increased PRR of the replicative gaps via TLS or TS, this could possibly promote PARPi resistance (Figure 3B). However, further investigation is necessary to fully understand the ramifications of this model. For example, it is complicated to truly uncouple gaps from HR and fork protection, making it difficult to form definitive conclusions. Furthermore, it is likely that a combination of HR, fork protection and gap suppression deficiencies contributes to increased sensitivity to chemotherapies, which would challenge a model in which one genotoxic phenotype is responsible for sensitizing cells to PARPi. We recently showed that loss of MED12 confers PARPi resistance in BRCA-deficient cells. Mechanistically, we found MED12 depletion in BRCA1- or BRCA2-knockout cells restored HR, elicited RF protection and conferred a decrease in ssDNA gap accumulation (121). While what exactly is contributing to these resistance phenotypes remains unclear, it illustrates that chemosensitivity can be complex and the driving forces behind sensitivity might be context dependent.

## ADDRESSING PARPi RESISTANCE IN THE CLINIC

While treatment of patients with PARPi in the clinic has been successful in cases of BRCA-deficient cancers, acquired resistance remains an obstacle in achieving durable responses. Furthermore, roughly 40% of patients harboring BRCA-mutant cancers never respond to PARPi (5). This highlights a clear need for alternative strategies for overcoming PARPi resistance in the clinic. There are currently several drugs being investigated to restore sensitivity to PARPi, some of which are discussed below. In studying these novel approaches, it will be important to take into consideration the therapeutic window of these drugs as well as their potential cancer-type selectivity.

**Figure 4.**
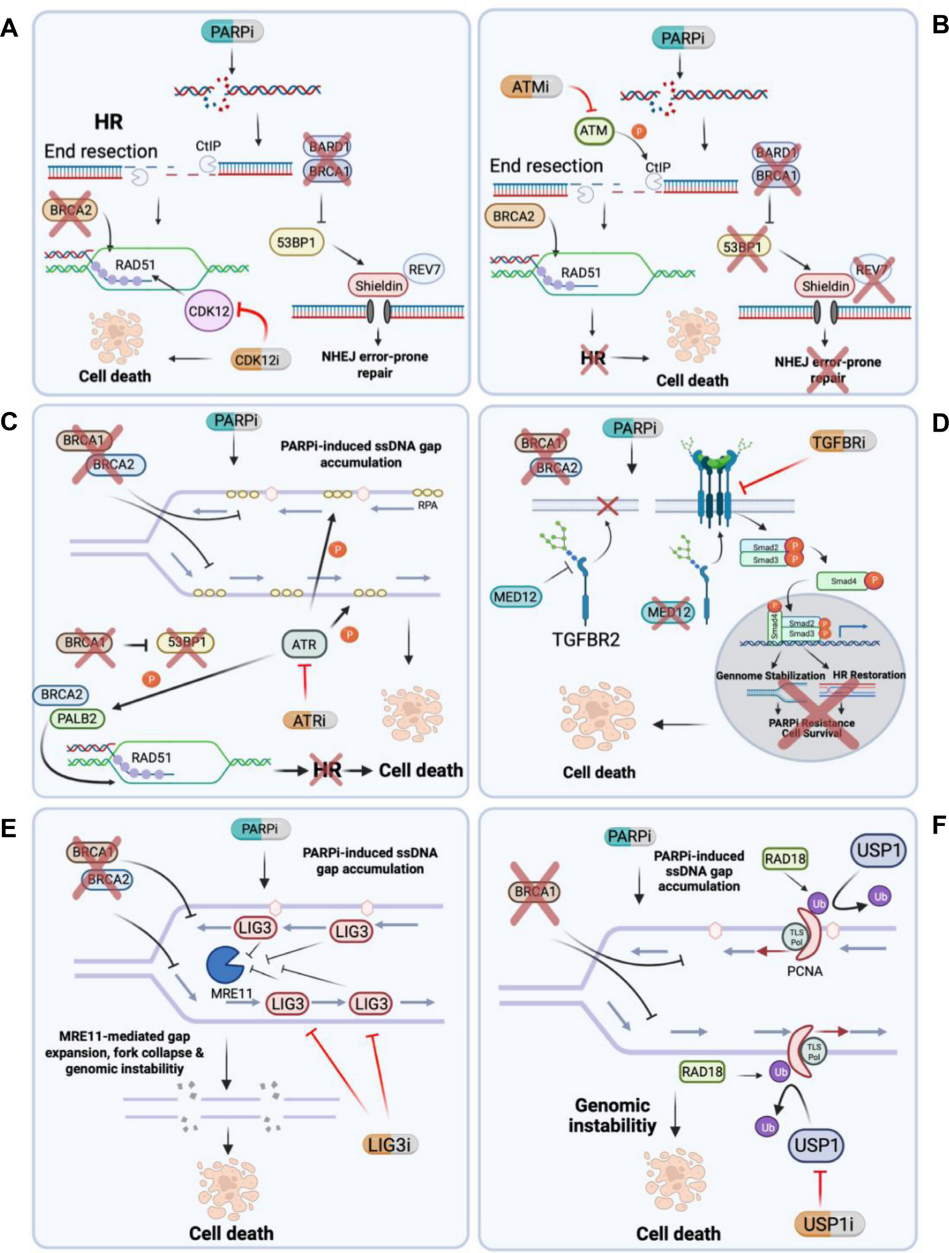
Hypothetical models for addressing PARPi resistance in the clinic. (**A**) CDK12 is known to regulate various DNA damage response genes and its inhibition can re-sensitize PARPi-resistant cells in a manner that leads to an increase in RAD51 levels. (**B**) ATM is critical for DNA damage response, including phosphorylation of CtIP. ATM inhibition can restore PARPi sensitivity in BRCA/NHEJ double-deficient cells, possibly by preventing the reinstated CtIP-mediated end resection necessary for HR, leaving the cells HRD. (**C**) ATR is important for sensing ssDNA, activating damage response and phosphorylating RPA. Inhibition of ATR abolishes PARPi resistance, possibly by preventing proper RPA phosphorylation and ssDNA gap suppression. Alternatively, ATR-mediated phosphorylation of PALB2 is important for RAD51 loading in BRCA1/53BP1 double-deficient cells; therefore, inhibition of ATR may reverse restoration of HR by preventing PALB2 loading of RAD51, leading to PARPi sensitization. (**D**) Loss of MED12 confers PARPi resistance to BRCA-deficient cells via activation of the TGFβ pathway and subsequent downstream signaling of this pathway including activation of SMAD proteins, ultimately leading to HR restoration and genome stabilization. This resistance can be overcome by inhibiting TGFBR in combination with PARPi. (**E**) LIG3 inhibition can re-sensitize PARPi-resistant BRCA1-deficient cells by allowing MRE11-mediated gap expansion and degradation to promote genomic instability and cell death. (**F**) USP1 carries a critical role in deubiquitinating PCNA. In BRCA1-deficient cells displaying RF stabilization and PARPi resistance, USP1 inhibition can re-establish PARPi sensitivity by promoting prolonged PCNA ubiquitination and subsequent TLS polymerase overloading at the site of ssDNA gaps, leading to genomic instability and cell death. Created with BioRender.com.

The cyclin-dependent kinase CDK12 has been shown to be a key regulator in various DNA damage response genes and its loss has been shown to compromise HR (122–126). A recent study showed that the non-specific CDK inhibitor dinaciclib possesses potent inhibitory activity against CDK12, and treatment of BRCA1-mutant TNBC cells with *de novo* or acquired resistance to PARPi and displaying elevated RAD51 with this drug re-sensitized them to PARPi (122). Furthermore, treatment of BRCA-WT TNBC cells with dinaciclib compromised HR and sensitized them to PARPi treatment. More specifically, dinaciclib treatment led to a decrease in RAD51 transcript levels, protein levels and foci formation, which possibly explains the sensitization observed. As discussed earlier, increased RAD51 levels have been shown to promote PARPi resistance by restoring HR (17,40,42); therefore, it is plausible that conferring a decrease in RAD51 would be sufficient to re-establish sensitivity (Figure 4A). These findings could be phenocopied using CRISPR-mediated knockout of CDK12 suggesting that the PARPi sensitivity observed using dinaciclib is due to CDK12 inhibition. These data suggest that patients who fail to respond to PARPi could be sensitized or re-sensitized by combining PARPi with a CDK12 inhibitor. Indeed, a phase 1 clinical trial (NCT01434316) is ongoing to study the safety and efficacy of using dinaciclib with the PARPi veliparib in patients not responding to treatment (122).

DNA damage response factors ATM and ATR have also been implicated in reversing PARPi resistance. It was previously shown that upon DNA damage, ATM kinase activity is essential to phosphorylate and activate CtIP and mediate end resection (10,127,128), and combining PARPi with an ATM inhibitor (ATMi) increases genomic instability (10). Using BRCA1-mutant PDX models that display PARPi resistance due to loss of 53BP1 or REV7, it was shown that combination treatment of the PARPi olaparib with the ATMi AZD0156 restores sensitivity (Figure 4B). Surprisingly, this did not lead to a significant decrease in HR as RAD51 foci formation was only marginally affected; however, cells with combined treatment did show a significant increase in γH2AX signal, suggesting increased replication stress. A phase 1 clinical trial evaluating the combination of AZD0156 with olaparib is currently ongoing (NCT02588105) (42).

PARPi have been shown to increase ATR/CHK1 signaling (129). Using BRCA-mutant cell lines with derived PARPi resistance, it was shown that ATR/CHK1 signaling increases even more compared to PARPi-sensitive BRCA-mutant parental cell lines, suggesting that the resistant cell lines rely heavily on ATR signaling for survival. In line with this, combined treatment of an ATR inhibitor (ATRi) with PARPi in PARPi-resistant cells increased replication stress and apoptosis and reversed the observed resistance. Using PDX mouse models with derived PARPi resistance, combined treatment with ATRi led to tumor regression, suggesting that this efficacy translates to *in vivo* models as well (130). Furthermore, it has been shown that combined PARPi treatment with ATRi dramatically increases sensitivity in PARPi-resistant, BRCA1-deficient BR5-R1 cells (79,131). This combined treatment in BR5-R1 cells was shown to significantly increase ssDNA gap formation, which could explain the increased sensitivity observed (79). A primary activator of ATR is the presence of RPA-bound ssDNA, allowing ATR-mediated phosphorylation of RPA (86,132). It is possible that loss of ATR leads to a decrease in RPA phosphorylation, which promotes the persistence of gaps and PARPi sensitivity (Figure 4C). Indeed, it was previously shown that ATR-mediated RPA phosphorylation prevents ssDNA gap accumulation in the presence of replication stressing agents (132). Alternatively, PALB2, a partner of BRCA2 important for loading of RAD51 during HR, has also been shown to be a target of ATR phosphorylation (131,133,134). It was revealed that BRCA1/53BP1 double-deficient cells display restored HR that is dependent on ATR, and treatment of these cells with PARPi in combination with ATRi significantly reduces RAD51 foci and promotes sensitivity, possibly due to a loss of PALB2 phosphorylation (131). Furthermore, ATR-mediated phosphorylation of RPA is also critical for binding of PALB2 to ssDNA (135) suggesting that ATRi-mediated PARPi sensitivity is complex but RPA phosphorylation might be at the center of it. Taken together, this work highlights a strategy for overcoming PARPi resistance both *in vitro* and *in vivo*. An ongoing phase 1 clinical trial (NCT04491942) is investigating the combined use of cisplatin with the ATRi BAY1895344 in patients with advanced breast carcinomas.

By employing a genome-wide CRISPR knockout PARPi resistance screen in BRCA2^KO^ HeLa cells, we recently identified loss of MED12 as a top hit for conferring resistance to PARPi (121). We determined that the resistance mediated by MED12 was related to its ability to post-transcriptionally modify TGFβ receptor TGFBR2 by preventing proper glycosylation and cell surface expression of the receptor, an already established role of MED12 (136). As a result, loss of MED12 promotes cell surface expression of TGFBR2 and activation of this pathway was found to underlie the PARPi resistance observed upon loss of MED12 in BRCA-deficient cells. Direct activation of the TGFβ pathway was able to restore HR and RF protection in BRCA-deficient cells, potentially explaining the resistance observed. Whether direct activation of the TGFβ pathway is able to suppress ssDNA gap formation remains an open question. Importantly, we were able to partially restore PARPi sensitivity in BRCA-deficient cells depleted of MED12 by treating with a TGFBR inhibitor (Figure 4D). While further investigation is necessary to fully understand MED12–TGFβ-mediated PARPi resistance mechanistically, our work identifies a new biomarker for resistance to PARPi and a potential novel strategy for combating this resistance (121).

In a recent study using a genetic screen to identify genes whose loss restores PARPi sensitivity to BRCA1/53BP1 double-deficient cells, LIG3 was identified as an enhancer of PARPi toxicity. This increase in PARPi sensitivity was shown to be independent of 53BP1 status but dependent on BRCA1 deficiency. Mechanistically, this sensitivity was found not to be related to decreased HR or LIG3’s role in POLθ-mediated end joining but instead was due to an increase in MRE11-driven post-replicative ssDNA gaps (Figure 4E). Importantly, a LIG3-mediated increase in PARPi toxicity translated *in vivo* using BRCA1-deficient organoids with shRNA depleted LIG3 transplanted into mouse mammary tissue, further supporting a therapeutic approach to PARPi resistance by inhibiting LIG3 (137). However, whether pharmacological inhibition of LIG3 would be feasible in patients remains unclear. While nuclear LIG3 is not essential for viability, loss of mitochondrial LIG3 is embryonic lethal in mice; therefore, toxicity may be an obstacle. Inhibitors specifically targeting nuclear LIG3 would likely be necessary (137,138).

Finally, the deubiquitinase USP1 has been implicated in DNA damage response as it has been shown to deubiquitinate PCNA (139,140). Because of this, it is an attractive target for cancer therapy (141). Expression of USP1 has been found to be enriched in BRCA1-deficient breast cancers due to its promotion of RF protection. This is likely because of the role USP1 has in regulating FANCD2. Not surprisingly, BRCA1 and USP1 are synthetically lethal. Treating BRCA1-deficient cells displaying PARPi resistance mediated by RF protection with the USP1 inhibitor ML323 re-sensitized these cells to PARPi. Interestingly, BRCA1-deficient cells displaying restored HR via 53BP1 silencing and subsequent PARPi resistance were not sensitive to ML323 suggesting that USP1 inhibition can overcome PARPi resistance in a subset of BRCA1-deficient cells displaying RF stabilization (142). Mechanistically, it was proposed that loss of USP1 leads to accumulation of RAD18-dependent monoubiquitinated PCNA, and persistent loading of low-fidelity TLS polymerases (142) (Figure 4F). Indeed, there is an ongoing phase 1 clinical trial (NCT05240898) investigating the use of KSQ-4279, a USP1 inhibitor, alone and in combination with PARPi.

## CONCLUDING REMARKS

Significant advances have been made in understanding the intricate nuances of PARPi sensitivity and resistance. One of the biggest limitations that remains is that large datasets of BRCA1- and BRCA2-mutant cancer patients treated with PARPi are not yet broadly available. Access to such datasets would allow for parallels to be drawn between what is observed *in vitro* and what is seen in patients treated with PARPi. Comparing the genetic profiles of a given patient’s tumor with their response to PARPi therapy would eventually allow a more precision medicine-based approach to treating cancer patients. While much work has been done to unravel the HR and RF stabilization-based mechanisms of resistance to PARPi, the recent shift in the field to ssDNA gaps as a determinant genotoxic lesion indicates that additional investigation is needed to fully understand these defining genotoxic lesions and what is truly at the core of PARPi sensitivity. Ongoing clinical trials to investigate the efficacy of combining PARPi with other novel targeted inhibitors show that new research findings are quickly translating from bench to bedside and provide hope that future cancer patients will benefit from a more targeted and durable approach to treatment.

## DATA AVAILABILITY

No new data were generated or analyzed in support of this research.
